# Editorial: Photon counting CT technology in cardiovascular imaging

**DOI:** 10.3389/fcvm.2025.1641175

**Published:** 2025-06-27

**Authors:** Bálint Szilveszter, Ákos Varga-Szemes, Amir Pourmorteza, Fides R. Schwartz

**Affiliations:** ^1^ Department of Radiology, Heart and Vascular Center, Semmelweis University, Budapest, Hungary; ^2^ Department of Radiology and Radiological Science, Medical University of South Carolina, Charleston, SC, United States; ^3^ Department of Radiology and Department of Imaging Sciences and Biomedical Informatics, Emory University School of Medicine, Atlanta, GA, United States; ^4^ Center for Advanced CT Translation and Innovation, Department of Radiology, Brigham and Women’s Hospital, Boston, MA, United States

**Keywords:** photon-counting CT, coronary artery disease, calcium score, phantom study, pericoronary adipose tissue (PCAT)

**Editorial on the Research Topic**
Photon counting CT technology in cardiovascular imaging

Since its clinical introduction in 2021, photon-counting detector CT (PCD-CT) has demonstrated significant advantages in the evaluation of cardiovascular disease, with most supporting evidence to date derived from the only commercially available whole-body dual-source PCD-CT system (1). This technology inherently provides spectral imaging capabilities for all scans, enhances spatial resolution, and reduces electronic noise (2–6). These improvements translate into better detection of stenosis, fewer referrals for invasive coronary angiography due to superior image quality and reduced calcium blooming artifacts—especially when using ultra-high-resolution modes—compared to conventional energy-integrating detector CT (EID-CT). However, whether PCD-CT can also improve the quantitative assessment of coronary plaques remains an open question. This summary presents recent findings on the application of PCD-CT for advanced cardiac evaluation.

Mergen et al. investigated the impact of ultra-high-resolution CT angiography on the quantitative characterization of coronary plaques. Reconstructions using the Bv40 kernel with a slice thickness of 0.6 mm were used as the reference standard. Using different kernel and slice thickness settings, they found substantial changes between the standard vs. ultra-high-resolution reconstructions using a 0.2 mm slice thickness and the Bv64 kernel (*p* < 0.001). The findings of this study highlight the importance of determining the optimal kernel settings—potentially validated through invasive methods—and understanding the impact of using different reconstruction parameters, as these not only affect the total plaque volume but also alter the assessed plaque composition. The proportion of calcified plaque component was 85.1% in standard reconstructions but dropped to 75.2% with ultra-high-resolution imaging, while lipid-rich plaque component increased to 6.7%. These results suggest that utilizing ultra-high-resolution PCD-CT reduces blooming artifacts for improved plaque delineation and characterization (3, 7).

Vascular inflammation plays a key role in the development of atherosclerosis and the rupture of atherosclerotic plaques, ultimately leading to adverse events (8). Kahmann et al. explored the relationship between pericoronary adipose tissue (PCAT) composition and coronary artery disease (CAD) in patients with plaques in the left coronary artery. PCAT has recently emerged as a promising non-invasive marker of vascular inflammation as assessed by CT, although its characteristics can vary widely depending on patient- and imaging-related factors. This analysis revealed that certain texture features significantly distinguished CAD patients from non-CAD individuals. Similar texture changes were also found in the right coronary artery (RCA), suggesting a systemic CAD effect on PCAT. These findings imply that PCAT texture alterations can be detected before plaque formation, offering potential for early CAD risk assessment and highlighting the value of advanced imaging techniques in risk stratification and management.

A recently published study by Kahmann et al. applied radiomics texture analysis on PCD-CT to detect subtle tissue differences associated with hypercholesterolemia, offering a potential imaging biomarker for the future. Six radiomic features differentiated those with and without hypercholesterolemia independently of coronary calcification or stenosis severity. Patients with hypercholesterolemia exhibited higher average PCAT attenuation (−97.1 HU vs. −100.0 HU) and increased dense tissue markers such as High Gray-Level Emphasis and High Gray-Level Run Emphasis. Conversely, High Gray-Level Zone Emphasis was lower in the hypercholesterolemia group, suggesting a more homogeneously dense tissue distribution, potentially reflecting low-grade inflammation. These results, validated in an independent cohort, indicate that PCAT radiomics on PCD-CT can detect subclinical tissue remodeling linked to lipid disorders, opening avenues for non-invasive early cardiovascular risk biomarkers beyond plaque morphology.

Coronary calcium scoring (CACS) is a widely used risk assessment tool guiding statin therapy, yet studies comparing PCD-CT to traditional EID-CT for CACS are limited. Wolf et al. directly compared CACS between PCD-CT and EID-CT in 23 patients and a phantom model. Using standard protocols, PCD-CT yielded systematically lower Agatston scores than EID-CT (median 174.3 vs. 218.2; mean bias −41.1). Phantom experiments showed PCD-CT more accurately estimated calcium volume (66.1% overestimation vs. 77.2% for EID-CT; *p* = 0.0015), with excellent correlation between modalities (*r* = 0.99). Simulations in a larger cohort suggested that PCD-CT scoring would reclassify about 5.25% of patients to a lower cardiovascular risk category. Overall, PCD-CT offers more precise physical quantification of coronary calcifications, but caution is needed when interpreting Agatston-based risk stratification across different scanner types.

While PCD-CT enhances diagnostic accuracy for coronary artery disease assessment, especially in high-risk populations, further studies are required to determine whether these imaging improvements translate into better long-term prognosis, more accurate risk stratification based on plaque characteristics, improved correlation with invasive plaque assessment, or enhanced evaluation of coronary stents.
